# Early gastric cancer with suspected brain metastasis arising eight years after curative resection: a case report

**DOI:** 10.1186/1756-0500-7-818

**Published:** 2014-11-19

**Authors:** Katsunobu Sakurai, Kazuya Muguruma, Akihiro Murata, Takahiro Toyokawa, Ryosuke Amano, Naoshi Kubo, Hiroaki Tanaka, Masakazu Yashiro, Kiyoshi Maeda, Masaichi Ohira, Kosei Hirakawa

**Affiliations:** Department of Surgical Oncology, Osaka City University Graduate School of Medicine, 1-4-3 Asahi-machi, Abeno-ku, Osaka City, Osaka, 545-8585 Japan

**Keywords:** Gastric cancer, Brain metastasis, Whole-brain radiation therapy

## Abstract

**Background:**

Brain metastasis from gastric cancer is fairly uncommon and prognosis is dismal. We report a case of early gastric cancer with brain metastasis arising 8 years after gastrectomy.

**Case presentation:**

A 78-year-old Japanese female presented to our hospital complaining of dizziness, 8 years after undergoing gastrectomy for gastric cancer. Histopathological type of the tumor was well-differentiated adenocarcinoma. Final TNM classification was T1b(sm1)N0M0, and pathological stage was IA. Further examination revealed a metastatic tumor in the cerebellum and multiple liver metastases. The brain metastasis was treated using radiotherapy and steroid. Systemic treatment for liver metastases was performed using the oral fluoropyrimidine drug S-1. Neurological symptoms decreased, enabling the patient to be discharged from hospital. However, chemotherapy was discontinued due to loss of appetite and general fatigue. She died 5 months after the diagnosis of brain metastasis due to progressive disease.

**Conclusions:**

Cases of brain metastasis arising 8 years after gastrectomy for early gastric cancer have rarely reported. Aggressive treatment for brain metastases may be effective for improvement of the damage to neurological function and quality of life.

## Background

Gastric cancer is one of the most common gastrointestinal tumors in Japan. Outcomes for patients with early gastric cancer have improved thanks to advances in diagnosis and treatment. However, patients with unresectable or recurrent gastric cancer still experience poor outcomes. Recurrence of gastric cancer often appears in the form of peritoneal dissemination, and liver metastases, lymph node recurrence and bone metastases are often seen.

The frequency of brain metastasis from gastric cancer is less than 1% [1–3]. Almost all brain metastases from gastric cancer are seen in advanced-stage disease with concurrent metastasis to other organs. Brain metastasis after curative gastrectomy for early gastric cancer appears extremely rare, and to our knowledge, there is no report of brain metastasis arising 8 years after curative operation for early gastric cancer. Palliative care is often performed for the patient with brain metastasis according to the other metastasis as terminal stage. However aggressive treatment such is warranted for brain metastases because of the damage to neurological function and quality of life (QOL). The present report describes a case with brain metastases after curative resection for early gastric cancer.

## Case presentation

**Figure 1:**
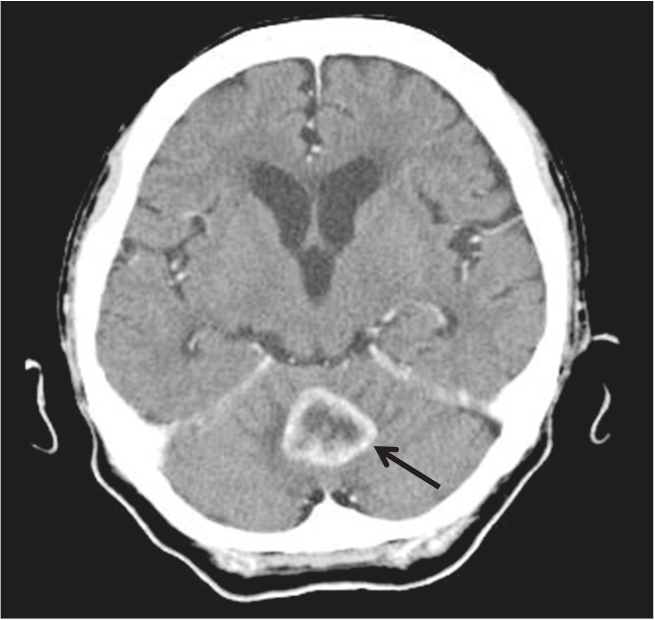
**Computed tomography (CT) of the brain shows a tumor (diameter, 2.5 cm) with ring enhancement in the cerebellum (arrow).**

**Figure 2:**
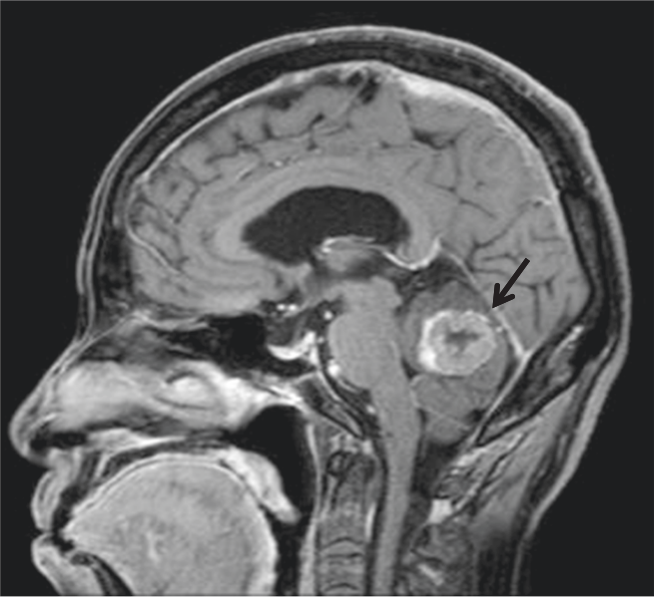
**Sagittal view T1-weighted magnetic resonance imaging (fat suppression) of the brain shows a 2.5-cm tumor with surrounding edematous change (arrow).**

**Figure 3:**
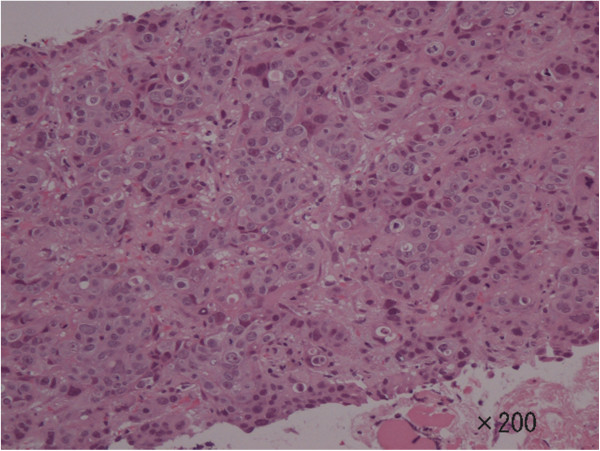
**Pathological examination of a biopsy specimen of the liver tumor shows metastatic adenocarcinoma that is poorly differentiated with solid and cord-like proliferation (hematoxylin and eosin, ×200).**

A 78-year-old Japanese female had been diagnosed with gastric cancer 8 years earlier and underwent distal gastrectomy. The tumor size was 45 × 45 mm, and no lymph node metastases were noted. Histological type of the tumor was well-differentiated adenocarcinoma. Final pathological TNM classification was T1b(sm1)N0M0, and the clinical stage was IA according to the Union for International Cancer Control criteria. Postoperative course was uneventful. After discharge, the patient underwent biannual follow-up with blood tests and abdominal computed tomography (CT). She received no adjuvant chemotherapy. She was referred to our hospital complaining of dizziness and an inability to walk. Brain CT revealed a tumor (diameter, 2.5 cm) with ring enhancement in the cerebellum (Figure 1). Abdominal CT revealed multiple liver metastases. No ascites or peritoneal dissemination was seen. Magnetic resonance imaging of the head showed an enhanced tumor with central necrosis in the cerebellum and surrounding edematous changes on T1-enhanced imaging (fat suppression) (Figure 2). Endoscopy and colonoscopy did not detect any recurrent lesions in the gastrointestinal tract. Tumor marker levels were as follows: carcinoembryonic antigen, 6.0 ng/ml; and carbohydrate antigen 19–9, 19 U/ml. Microscopic examination of hematoxylin and eosin (HE)-stained percutaneous biopsy specimens of the liver metastasis revealed adenocarcinoma (Figure 3). Increased uptake was seen in the liver tumors and para-aortic lymph nodes (standardized uptake values: vermis cerebellum, 6.7; liver, 5.5-6.2; and para-aortic lymph node, 2.2) on ^18^ F-fluorodeoxyglucose-enhanced positron emission tomography. We thus diagnosed the liver and brain tumors as metastases derived from gastric cancer.

**Figure 4:**
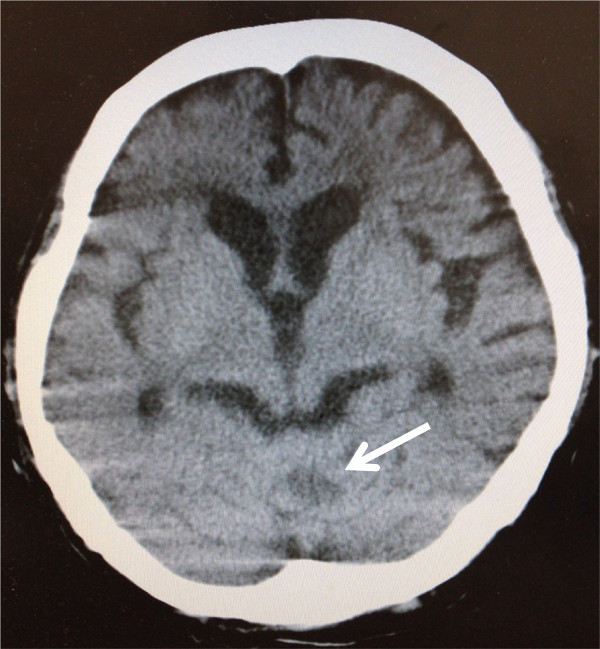
**At 10 weeks after radiotherapy, CT shows a tumor reduced in size (arrow).**

Radiotherapy for brain metastasis was performed in conjunction with corticosteroid administration (30 Gy for whole brain, including metastasis). At 10 weeks after radiotherapy, the metastatic brain tumor had decreased in size (Figure 4). Clinical symptoms decreased markedly after radiotherapy, enabling her to be discharged from hospital. She subsequently received the oral fluoropyrimidine drug S-1 for the treatment of liver and lymph node metastases. The regimen of S-1 consisted of six-week cycles in which, 100 mg of oral S-1 per body per day was given for four weeks and no chemotherapy was given for the following two weeks. However, chemotherapy was discontinued after two courses due to fatigue and loss of appetite. The patient’s performance status subsequently deteriorated, and she died 5 months after the diagnosis of brain metastasis.

### Discussion

In Japan, gastric cancer is the most common digestive cancer, and the incidence of gastric cancer in Japan is among the highest in the world (62.0 per 100,000 men, 26.1 per 100,000 women) [4]. The outcomes of surgical treatment for early gastric cancer (EGC) are generally considered satisfactory. However, a small percentage of patients experience disease recurrence. Sano *et al.* estimated a recurrence rate for EGC of at least 1.9% by combining the data from 20 reports in the literature [5]. On the other hand, advanced gastric cancer mostly recurs as peritoneal metastasis and often recurs in the lymph nodes, liver and bones. Brain metastases from gastric cancer are exceedingly rare. York *et al.* analyzed 3320 gastric cancer patients treated over a 40-year period and identified brain metastases in only 24 patients (0.7%) [1]. Kasakura *et al.* identified brain metastases in only 11 of 2322 patients (0.47%) [6]. These cases involved simultaneous or metachronous metastases from advanced stage disease. To our knowledge, there is no report of brain metastasis arising 8 years after curative operation for early gastric cancer as the present case.

It is not clear the mechanism of metastasis to liver and brain 8 years after curative resection. These metastases are hematogenous and might indicate that the cancer is a systemic disease, not local disease. It has been pointed out cancer cells are circulating in the peripheral blood from an early stage. The presence of peripheral blood circulating tumor cells (CTCs) is a predictor of relapse or poor prognosis and has documented even in early stage colon cancer or breast cancer [7–9]. Uen *et al.* investigated preoperative and postoperative CTCs in patients with stage I-III colorectal cancer who underwent curative resection; preoperative CTCs were detected in 33.3% of patients with stage I disease, and postoperative CTCs were detected in 6.0% of patients with stage I disease [8]. We speculate that post operative CTCs are present in patients with early gastric cancer undergoing R0 resection and that malnutrition or immune compromise promotes the proliferation and metastasis of CTCs, thereby increasing the risk of tumor recurrence.

Therapy for metastatic brain tumor includes surgical resection, radiation and chemotherapy or a multidisciplinary approach that combines these modalities. Stereotactic radiosurgery, such as cyber knife or gamma knife, has become the mainstay of therapy for metastatic brain tumors [10]. However, the response to treatment is poor among patients with brain metastasis from gastric cancer. Most brain metastases from gastric cancer are detected in the advanced stages, with metachronous or simultaneous metastasis to other organs [1, 6]. Therefore palliative care was performed in many cases. However, aggressive treatment should be undertaken to improve the neurological symptoms of brain metastasis if progression of metastases in other organs can be controlled. In our case, since performance status (PS) was poor, we judged there was no surgical indication. Therefore we selected whole brain radiation therapy. As a result, her neurological function has recovered dramatically by radiation therapy. From our experience, we consider that radiation therapy is useful for the brain tumor from gastric cancer in inoperable case.

The mean interval from gastrectomy to the diagnosis of brain metastasis has been reported as 9.0-9.6 months [1, 6]. Mean survival for patients with brain metastasis from gastric cancer is approximately 2 months, significantly less than the mean survival among patients with brain metastasis from breast cancer (5.6-6.8 months) [11, 12] or lung cancer (6.9-7.8 months) [13, 14]. York *et al.* reported that median survival time in patients who received whole-brain radiation therapy (WBRT) in conjunction with corticosteroids was 9 weeks after diagnosis of brain metastasis, and median survival in patients who underwent surgical resection of brain metastases in addition to WBRT and steroids was 54 weeks [1]. Kasakura *et al.* reported that survival rates were highest for the resection group, followed by the chemoradiotherapy group and then the no-treatment group [6]. Median survival for these groups was 24 weeks (n = 3), 7.4 weeks (n = 4) and 2.8 weeks (n = 4), respectively.

The most common presenting symptoms for brain metastasis from gastric cancer are weakness (67%), headache (42%), balance/gait abnormalities (42%), and nausea/emesis (38%) [1]. Kasakura *et al.* reported that all patients with brain metastasis had stage ≥ III cancer, all had lymphovascular invasion or lymph node metastasis, and many had metastases to other organs [6]. York *et al.* reported that all patients with brain metastases from gastric cancer showed concurrent systemic metastasis to the lymph nodes, bones, liver and lungs [1]. Nomura *et al.* reported a case of brain metastasis from submucosal gastric carcinoma, but that patient had stage IV cancer accompanied by liver metastasis [15].

No factors that can predict brain metastases have yet been identified. Alpha-fetoprotein (AFP)-producing gastric cancer with brain metastasis has been reported [16, 17] and AFP may thus be useful as a predictive factor for brain metastasis.

We often detect metastases to other organs at the same time as diagnosis of brain metastasis from gastric cancer. Brain tumor from gastric cancer is thus the end picture of multi-organ metastasis and carries a poor prognosis. Cause of death is often metastasis, though metastasis to the brain is rare. Because neurological symptoms associated with metastatic brain tumors significantly reduce QOL for the patient, aggressive treatment of symptoms is very important to allow the patient to spend the rest of their life in a meaningful way.

In our case, radiotherapy was treatment of the brain tumor and S-1 was treatment of the liver tumor. A combination of cisplatin and S-1 chemotherapy is a standard regimen for unresectable gastric cancer in Japan [18], but her PS was poor. Therefore we selected S-1 monotherapy. Effective chemotherapy for brain tumor is not known well. Intravenously administered agents do not generally cross the cerebral parenchyma due to the blood–brain barrier (BBB). However Kitayama *et al.* reported a case in which paclitaxel therapy proved effective against gastric cancer with brain metastasis [19]. Kokufu *et al.* also reported the usefulness of paclitaxel for brain metastasis from breast cancer [20].

A major protein constituent in the BBB is P-glycoprotein (P-gp). Gerstner *et al.* reported that immunochemical P-gp expression in the neovasculature of metastatic brain tumors was less than that of gliomas and suggested that metastatic brain tumors with low P-gp expression might be more permeable to natural product chemotherapy drugs than gliomas [21]. Fine *et al.* reported that the median concentration of paclitaxel was 2.46-fold higher in the periphery of metastatic brain tumors as compared with gliomas suggesting decreased BBB function likely secondary to decreased expression of P-gp in neovascular endothelial cells of metastatic brain tumors [22]. We consider that since the BBB is damaged in patients with marked brain metastasis, intravenously administered agents may be able to cross the cerebral parenchyma. In addition, paclitaxel is known to act as a radiosensitizer to increase the radiosensitivity of tumor cells. Paclitaxel chemotherapy in combination with radiation can thus be expected to offer a useful treatment for brain metastasis. Effect of chemotherapy for brain tumor should be examined further.

## Conclusion

Brain metastases arising after gastrectomy for early gastric cancer is extremely rare. Palliative care is often chosen for brain metastasis according to the other metastasis as terminal stage. However, aggressive treatment for brain tumors, such as radiotherapy or chemotherapy, appears useful for improving neurological function and allowing the patient to spend the rest of their life in a meaningful way. Patients with a history of gastric cancer and neurological symptoms should be examined while keeping the possibility of brain metastasis in mind, because brain metastasis can arise even in long-term follow-up after curative resection for early gastric cancer.

## Consent

Written informed consent was obtained from the patient’s next of kin for publication of this case report and any accompanying images. A copy of the written consent is available for review by the Editor-in-Chief of this journal.

## Abbreviations

QOL: Quality of life
CT: Abdominal computed tomography
HE: Hematoxylin and eosin
EGC: Early gastric cancer
CTCs: Circulating tumor cells
PS: Performance status
WBRT: Whole-brain radiation therapy
AFP: Alpha-fetoprotein
BBB: Blood–brain barrier
P-gp: P-glycoprotein.
